# Safety and PCR monitoring in 161 semi-immune Kenyan adults following controlled human malaria infection

**DOI:** 10.1172/jci.insight.146443

**Published:** 2021-09-08

**Authors:** Melissa C. Kapulu, Patricia Njuguna, Mainga Hamaluba, Domtila Kimani, Joyce M. Ngoi, Janet Musembi, Omar Ngoto, Edward Otieno, Peter F. Billingsley, the Controlled Human Malaria Infection in Semi-Immune Kenyan Adults (CHMI-SIKA) Study Team

**Affiliations:** 1 Centre for Geographic Medicine Research, Coast, Kenya Medical Research Institute-Wellcome Trust Research Programme, Kilifi, Kenya.; 2 Centre for Tropical Medicine and Global Health, Nuffield Department of Medicine, University of Oxford, Oxford, United Kingdom.; 3 Sanaria, Rockville, Maryland, USA.; 4 The CHMI-SIKA Study Team is detailed in Supplemental Acknowledgments.

**Keywords:** Clinical Trials, Infectious disease, Malaria, Parasitology

## Abstract

**BACKGROUND:**

Naturally acquired immunity to malaria is incompletely understood. We used controlled human malaria infection (CHMI) to study the impact of past exposure on malaria in Kenyan adults in relation to infection with a non-Kenyan parasite strain.

**METHODS:**

We administered 3.2 × 10^3^ aseptic, purified, cryopreserved *Plasmodium falciparum* sporozoites (Sanaria PfSPZ Challenge, NF54 West African strain) by direct venous inoculation and undertook clinical monitoring and serial quantitative PCR (qPCR) of the *18S* ribosomal RNA gene. The study endpoint was met when parasitemia reached 500 or more parasites per μL blood, clinically important symptoms were seen, or at 21 days after inoculation. All volunteers received antimalarial drug treatment upon meeting the endpoint.

**RESULTS:**

One hundred and sixty-one volunteers underwent CHMI between August 4, 2016, and February 14, 2018. CHMI was well tolerated, with no severe or serious adverse events. Nineteen volunteers (11.8%) were excluded from the analysis based on detection of antimalarial drugs above the minimal inhibitory concentration or parasites genotyped as non-NF54. Of the 142 volunteers who were eligible for analysis, 26 (18.3%) had febrile symptoms and were treated; 30 (21.1%) reached 500 or more parasites per μL and were treated; 53 (37.3%) had parasitemia without meeting thresholds for treatment; and 33 (23.2%) remained qPCR negative.

**CONCLUSION:**

We found that past exposure to malaria, as evidenced by location of residence, in some Kenyan adults can completely suppress in vivo growth of a parasite strain originating from outside Kenya.

**TRIAL REGISTRATION:**

ClinicalTrials.gov NCT02739763.

**FUNDING:**

Wellcome Trust.

## Introduction

Humans become immune to *Plasmodium falciparum* malaria following repeated exposure (1). Immunoepidemiological studies show associations between immune responses and protection against malaria in the field (2), but their interpretation is complicated by heterogeneity of exposure (3). Furthermore, malaria is genetically diverse, and vaccines that protect against heterologous parasites are required for sustained public health impact. Exposure in the field is usually with parasites of unknown genotype, further complicating inferences on protective immunity. An experimental design with controlled exposure to parasites, such as the controlled human malaria infection (CHMI) model, is needed for greater confidence in inferring causality and to directly test for heterologous immunity.

Human infection studies in which investigators intentionally infect healthy volunteers have been used to understand pathogenesis, immunity, and genetic resistance to infection and to measure the efficacy of drugs and vaccines (4). The majority of CHMI studies performed to date have used *P*. *falciparum*–infected mosquitoes to assess vaccine-induced efficacy in nonmalaria endemic populations. The availability of aseptic, purified, cryopreserved *P*. *falciparum* sporozoites (Sanaria PfSPZ Challenge), administered by needle and syringe has facilitated CHMI studies among nonendemic and endemic populations (5–7). An intravenous dose of 3.2 × 10^3^ PfSPZ leads to an established infection with exponential in vivo parasite growth in 100% of volunteers from malaria nonendemic areas (8, 9).

Sanaria PfSPZ Challenge has now been used in several malaria endemic populations. A total of 150 volunteers in 4 malaria-endemic countries (Gabon, The Gambia, Kenya, and Tanzania) have been enrolled in published CHMI studies using Sanaria PfSPZ Challenge to either assess vaccine efficacy or infectivity (6, 7, 10–12). These studies have been used to evaluate vaccine efficacy (12); naturally acquired immunity (6, 7, 10); and innate resistance to infection (11). A further 300 volunteers have been or will be enrolled in vaccine efficacy CHMI trials in West and East Africa over the next 2–3 years (13). Most of the volunteers in these CHMI studies were from urban areas (i.e., areas with limited prior malaria exposure). Nevertheless, this limited prior exposure led to CHMI outcomes in these individuals that were different from those seen in nonendemic regions (6, 7, 10, 11). Ten of forty-three volunteers did not develop positive blood smears for malaria parasites in studies in Tanzania (6) and Gabon (11), whereas 100% of volunteers in nonendemic areas develop positive blood smears. In a vaccine efficacy study conducted in Tanzania (12), infectivity detectable by blood smear microscopy in the control group was observed in 16 of 18 volunteers. In Kenya, Nairobi, 1 of 28 volunteers was negative by blood smear microscopy and showed minimal parasite growth by serial quantitative PCR (qPCR; ref. 7). In The Gambia, 17 of 19 volunteers developed infection detectable by microscopy, 1 was negative by microscopy but positive by qPCR, and another 1 was negative by qPCR (10).

For endpoint assessment in CHMI studies, the conventional method for monitoring parasitemia has been thick-blood smear microscopy, which does not detect low-density parasitemia (14). Molecular markers such as qPCR are substantially more sensitive (15) and, therefore, provided more information on parasite growth in the CHMI studies referenced above in Kenya and in The Gambia. This molecular marker–based approach has recently been qualified by the US Food and Drug Administration as a replacement for thick-blood smears in nonendemic CHMI studies (16).

To systematically explore qPCR outcomes following CHMI in semi-immune adults, we conducted the largest CHMI study to date, recruiting volunteers with previous exposure to malaria from specific rural areas in Kenya. As adults in malaria-endemic regions of Kenya are frequently found to be asymptomatic despite significant blood parasitemia (17), we adopted a relatively high threshold (500 parasites/μL) for the study endpoint. This threshold is higher than that used for CHMI in nonendemic areas (where thick-blood smear microscopy has been the criteria for treatment, equivalent to approximately 5–50 parasites per μL; ref. 15). However, we expected that parasites would be well tolerated among Kenyan adults, as the threshold is 5 times below the parasitemia associated with symptomatic malaria in Kenyan children (i.e., 2500 parasites/μL; ref. 17). We included criteria for treatment at lower parasite densities in the presence of important clinical symptoms.

The aim of this study was to investigate how the in vivo parasite growth in CHMI would be modified by preexisting immunity, where qPCR was used to quantify parasite growth and PfNF54 parasites were used for challenge. NF54 is a laboratory-adapted parasite line that is of West African origin and is genetically distinct from East African parasites (18). Moser et al. have recently shown that East African parasites (including parasite clinical isolates from Kenya) are genetically distant from NF54 parasites (18). Based on phylogenetic analysis, NF54 has been shown to cluster with West African parasites. The genetic distance between NF54 and East African parasites is similar to the distance between NF54 and South American parasites, and therefore, cross-protection against NF54 can be attributed to exposure to genetically distant parasites.

## Results

### Study design and study population.

**Figure 1:**
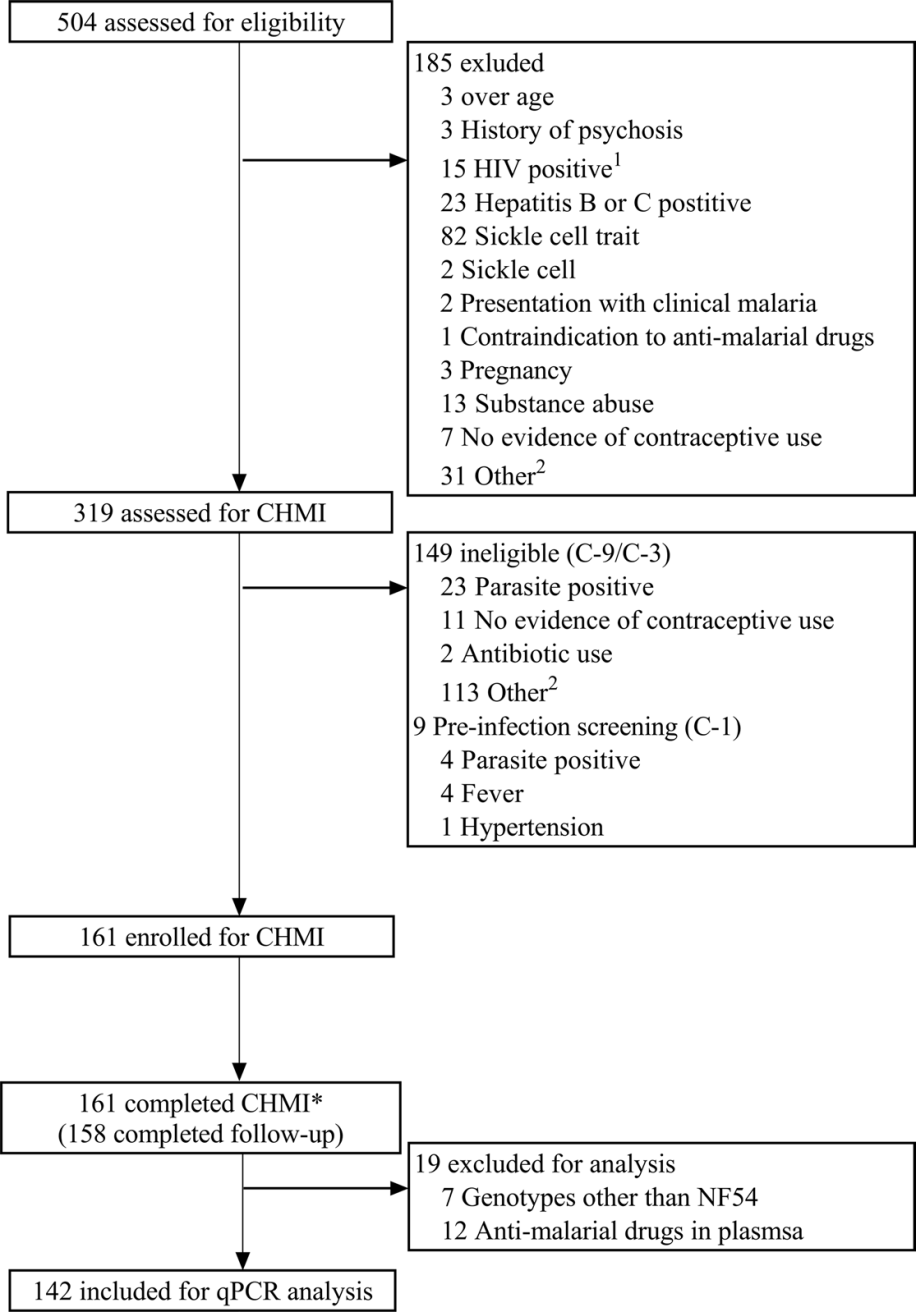
Study design and volunteer eligibility and enrollment for CHMI. ****^1^One volunteer was both HIV and hepatitis B positive. ^2^Volunteers were assessed in the fourth quarter of 2017 but were not enrolled for CHMI due to national security reasons. *Volunteers were deemed to have completed CHMI if they had received endpoint treatment. qPCR outcomes are presented for 142 volunteers after exclusions for parasite genotype and antimalarial drug levels.

Five hundred and four volunteers were recruited from Ahero, Kilifi North, and Kilifi South locations in Kenya and assessed for eligibility. Of these, 161 healthy volunteers were enrolled into 3 successive cohorts beginning in August 2016 (*n* = 37), February 2017 (*n* = 64), and February 2018 (*n* = 60), representative of volunteers from Ahero (*n* = 15), Kilifi North (*n* = 34), and Kilifi South (*n =* 112). All 161 volunteers were inoculated with 3.2 × 10^3^ PfSPZ of Sanaria PfSPZ Challenge NF54 by direct venous inoculation (DVI) and monitored for outcomes by clinical assessment and qPCR. All volunteers completed CHMI and were successfully treated and discharged from inpatient care (Figure 1). One hundred and sixty-one volunteers completed CHMI; they were considered to have completed CHMI if they if they had received endpoint treatment. Of these, 158 volunteers completed follow-up at day 35 after CHMI (day 90 for the 2016 cohort). Two volunteers moved away from the study area, and one was not available for follow-up visit(s) after CHMI was completed. Data from these 2 volunteers were included for analysis of CHMI outcomes. Seven volunteers were found to have non-PfNF54 strain parasites (Supplemental Data File 1; supplemental material available online with this article; https://doi.org/10.1172/jci.insight.146443DS1) and were excluded from further analysis; a further 12 volunteers were excluded on the basis of antimalarial drug concentrations (detected at time points 1 day before challenge [C-1] and 8 days after challenge [C+8], see below; Supplemental Data File 2). Thus, the safety analysis is reported for all 161 volunteers who underwent CHMI, and qPCR outcomes are described for 142 volunteers (Figure 1). The mean age at enrollment was 29.5 years old (SD 7.1 years) and most volunteers were male (70.2%; Table 1). The baseline characteristics of the 161 enrolled volunteers are shown in Table 1.

### Adverse events.

There were no serious adverse events reported. Adverse event reporting was divided into 3 separate time periods: (a) the first 6 days from day of administration of PfSPZ (days 1–7), when there were few grade 1 local adverse events relating to the injection site and some isolated unsolicited events; (b) day 8 after administration of PfSPZ up to the day of treatment, when systemic febrile events (e.g., headache followed by fever, myalgia, and sweating) became more common; and (c) the day of treatment to the exit from in-patient stay, when systemic febrile adverse events were most common (Table 2). Unsolicited adverse events reflected symptoms of febrile malaria, with a few additional events at low frequency. Solicited and unsolicited adverse events were all found to be mild (i.e., grade 1). The most common adverse event reported was headache, followed by fever, myalgia, and sweating. The most commonly used concomitant medications were antipyretics (Supplemental Table 2).

The most common abnormalities on safety blood tests during CHMI were grade 1 and 2 elevated alanine aminotransferase (ALT) and creatinine values, all of which resolved to within the normal range during the follow-up period (Supplemental Table 3). The cutoffs for ALT grade 1 were >80 to <200 IU/L and >55 to <138 IU/L, while cutoffs for grade 2 were 200 to <400 IU/L and 138 to <275 IU/L, respectively, for male and female volunteers. The cutoffs for creatinine grade 1 were >111 to 144 μmol/L and >92 to <119 μmol/L while grade 2 cutoffs were >144 to 199 μmol/L and >119 to <166 μmol/L, respectively, for male and female volunteers. ALT increases were more common in male compared with female volunteers (Supplemental Figure 1). Markedly reduced platelet counts (grade 4) were observed in 2 volunteers, as has previously been described in malaria (19). There were no signs of any bleeding tendency, consistent with previous reports of severe thrombocytopenia in malaria (19).

### Assessment of presence of antimalarial drugs.

Antimalarial drugs were detected in some volunteers (Table 3 and Supplemental Figure 2). Two volunteers had detectable concentrations of chloroquine (i.e., 3.6 ng/mL and 5 ng/mL, respectively) before CHMI, both at concentrations well below the reported minimum inhibitory concentration (MIC) of 67 ng/mL (20) (i.e., at 5 ng/mL plasma and 3.6 ng/mL plasma, respectively); one of these met criteria for treatment before day 22, the other did not. Twelve volunteers had lumefantrine concentrations above the reported MIC of 280 ng/mL plasma (21), none of them met criteria for treatment. A further 64 volunteers had concentrations below the MIC, of whom 43 met criteria for treatment before day 22, and 21 did not. Thus, among volunteers with detectable lumefantrine concentrations (albeit below the MIC), criteria for treatment were met slightly less often than among volunteers in whom lumefantrine was undetectable, 33.4% (25 of 73) versus 43.5% (33 of 76) (*P =* 0.25; Table 3).

One volunteer had high concentrations of both pyrimethamine and sulfadoxine and did not meet criteria for treatment. Two further volunteers had detectable concentrations of sulfadoxine without pyrimethamine, and both met criteria for treatment before day 22. There was no detectable artemether or dihydroartemisinin in any sample. The prevalence of detectable lumefantrine concentrations was higher in Kilifi South (62.5%, 70 of 112) compared with that in Kilifi North (20.6%, 7 of 34). Lumefantrine concentrations measured at the 2 independent laboratories were closely correlated (*r* = 0.93, *P <* 0.0001).

### Analysis of qPCR outcome following CHMI.

For further analysis of qPCR outcomes by location, we therefore excluded volunteers with lumefantrine concentrations above the MIC, and we excluded the volunteer with high pyrimethamine and sulfadoxine concentrations. Concentrations of chloroquine or of lumefantrine below the MIC were not strongly associated with outcome, and, therefore, we retained data from volunteers with these drug levels and those with undetectable drug levels. Thus, we included data from 142 volunteers for further analysis (Table 3). One volunteer requested early treatment at C+21 and, therefore, was missing qPCR data from C+22. The volunteer was qPCR negative up to day 21, and it was therefore assumed they would be negative at day 22, and their data were included in the final analysis.

**Figure 2:**
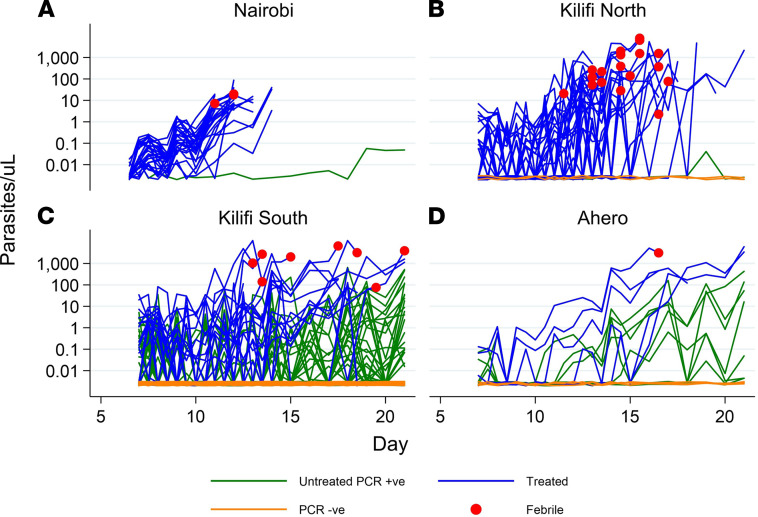
qPCR outcome based on volunteer location. Blood samples from C+8 (C+7.5 for Nairobi) onward after inoculation to determine parasitemia from (**A**) Nairobi (*n =* 27), (**B**) Kilifi North (*n =* 34), (**C**) Kilifi South (*n =* 93), and (**D**) Ahero (*n =* 15). Parasitemia was determined by asexual *18S* ribosomal RNA gene qPCR done in Kilifi. Blue lines represent individuals who required treatment and reached treatment threshold (reached DoT). Green lines represent individuals who did not meet criteria for treatment threshold but were qPCR positive. Orange lines represent individuals who were qPCR negative throughout monitoring. Red dots denote individuals who were febrile and met treatment criteria.

We combined our data from Kilifi and Ahero with data from a previous pilot CHMI study conducted in Nairobi (an urban area with no malaria transmission; ref. 7). The qPCR results over time in relation to location, outcome, and febrile status are shown in Figure 2. Heterogeneous patterns of parasite growth were observed over time with (a) some volunteers showing rapid and consistent growth; (b) others showing early parasite growth that subsequently appeared to be suppressed; (c) late growth that was inconsistent and did not reach the threshold criteria for treatment; or (d) negative qPCR results throughout the period of monitoring. Individual qPCR results for each volunteer are shown as Supplemental Figure 3, A–D, for the respective locations — Nairobi, Ahero, Kilifi North, and Kilifi South. In addition, individual qPCR results are shown for each respective successive cohort from 2016, 2017, and 2018 (Supplemental Figure 4).

Of the 142 volunteers who resided in Kilifi or Ahero (i.e., excluding Nairobi residents), 26 (18.3%) had febrile symptoms and were treated, 30 (21.1%) reached 500 or more parasites per μL and were treated, 53 (37.3%) had parasitemia without meeting thresholds for treatment, and 33 (23.2%) remained qPCR negative, 10 of these with no detectable antimalarial drug levels (Supplemental Table 4). Of the 28 volunteers previously included in CHMI in Nairobi, all experienced parasite growth, with 27 (96.4%) meeting the criteria for malaria diagnosis; 1 volunteer (3.6%) had parasitemia without meeting thresholds for treatment (7). Table 4 shows the distribution of these qPCR outcomes by location. Criteria for treatment were more frequently met among volunteers from Nairobi and Kilifi North and less frequently met for volunteers from Ahero or Kilifi South (Table 4).

In contrast, in the high transmission locations of Kilifi South and Ahero it was more common to observe volunteers that experienced limited parasite growth or with negative qPCR results. The presence of fever was more common in Kilifi North than in Kilifi South or in Ahero. Fever was less frequent in the Nairobi study, in which treatment was given based on microscopy (microscopy is positive at 50 parasites/μL by qPCR assay, ref. 14; but volunteers were treated at 500 parasites/μL blood in the present study). Similar results were seen after excluding all volunteers with detectable lumefantrine concentrations (Supplemental Table 4).

### PCR parasite detection comparisons.

A subset of samples from the 2018 enrollment cohort, a total of 120 samples corresponding to 20 volunteers from time points C+8 to C+10.5 (8 volunteers who were qPCR negative throughout in Kilifi and 12 other volunteers with positive readings), were shared with 2 external laboratories at Mahidol University (MU), Thailand, and at the University of Washington (UW), Seattle, Washington, USA, for their analytically sensitive qPCR and reverse-transcription PCR (RT-PCR) assays, respectively (15, 16, 22). There was close agreement of results from the Kilifi laboratory assay and those from MU (*r* = 0.65, *P <* 0.0001) and UW (*r* = 0.64, *P <* 0.0001) and between MU and UW assays (*r* = 0.79, *P <* 0.0001; Supplemental Figure 5). Of the 8 volunteers considered qPCR negative in the Kilifi assay, 5 had parasitemia detected at low levels in the MU assay, and 6 had low-level parasitemia in the UW assay (Supplemental Figure 6). Two of the eight volunteers were negative for all 3 assays, while 1 volunteer was positive for the UW assay but negative for the other 2 assays.

Finally, we reanalyzed the ALT elevations observed after CHMI according to qPCR outcomes and found that ALT elevations did not vary according to qPCR status. Volunteers who were qPCR negative for malaria parasites following CHMI had a median ALT of 46 IU/L (IQR 27–120 IU/L) at day 10, compared with a median ALT of 43 IU/L (IQR 26–80 IU/L) among those who were qPCR positive (*Z* = 0.79, *P =* 0.43; Supplemental Figure 7).

## Discussion

We found CHMI to be safe and well tolerated in the 161 volunteers enrolled in this study who were recruited from malaria-endemic regions of Kenya. Using qPCR, we show that outcomes following CHMI vary depending on previous history of malaria exposure. In malaria nonendemic locations, inoculation with the same dose of 3.2 × 10^3^ of Sanaria PfSPZ Challenge by DVI that was used in our study leads to infection and in vivo parasite growth in 100% of volunteers (8, 9). In our study with Kenyan volunteers, we observed in vivo parasite growth leading to parasite densities that meet treatment criteria in only 39.5% of our volunteers (i.e. 18.3% with symptoms plus 21.1% without). In 37.3% of the volunteers, we observed limited in vivo growth that was partially suppressed, such that treatment criteria were not met. In another 23.3% of the volunteers, we observed complete suppression of parasite growth, such that parasites were not detected by our qPCR assay. The challenge strain is genetically distant to East African parasites (18), implying that the complete suppression of parasite growth was the result of heterologous immunity.

We saw elevated levels of ALT following infection, which reverted to baseline following clearance of infection. Other CHMI studies in malaria-naive volunteers have reported elevated levels of liver enzymes, seen after mosquito bite and blood-stage challenge and in both *P*. *falciparum* and *P*. *vivax* (23–26). Furthermore, Dondorp et al. have observed increased liver enzymes following *P*. *falciparum* natural infection (27). Rises in liver enzymes appear more pronounced in naive populations. In our study, these increases were more pronounced in male volunteers (Supplemental Figure 1) but were not associated with any clinical indicators of serious illness.

This study provides the largest data set to date on CHMI outcomes in an adult population from a malaria-endemic area. The CHMI outcomes in participants from different locations clearly followed the underlying intensity of malaria exposure (i.e., with Nairobi appearing similar to nonendemic countries, and then Kilifi North, Kilifi South, and Ahero associated with increasing control of parasite growth). It is likely that prior malaria exposure rather than human genetic differences explains the observed variations in CHMI outcomes. In relation to more recently published CHMI studies in endemic populations (6, 7, 10, 11), we present here findings based on qPCR as the main readout for endpoint outcome as opposed to thick-blood smear, which has been traditionally used in other CHMI studies, and demonstrate, using more sensitive molecular methods for endpoint readout, that infection with a genetically distinct parasite has varying outcome, depending on the current and/or past exposure to malaria. Previous CHMI studies have enrolled volunteers with historic past exposure and little to no current exposure (6, 7, 10), and here, we show outcomes following CHMI that mirror the exposure patterns as evidenced by area of residence. Of note is the number of volunteers, who following infection, did not have febrile malaria, which also appears to be as a result of current and/or past exposure.

In this study, antimalarial drugs were detectable in a significant proportion of volunteers, in particular lumefantrine, which has a relatively long half-life (28). Thus, we excluded volunteers with baseline concentrations of lumefantrine above the reported MIC for *P*. *falciparum* (21). We analyzed lumefantrine concentrations below the MIC and found no association with CHMI outcome in these volunteers, who were therefore retained in the analysis. Furthermore, there was a similar pattern of results seen when we reanalyzed data after excluding all volunteers with detectable lumefantrine concentrations (Supplemental Table 4). Artemether-lumefantrine is widely available in Kenya and is the likely source of lumefantrine in our volunteers (29). Artemisinins were not detected in any volunteers, and these drugs have a very short half-life (30). The observed concentrations of lumefantrine were low or undetectable. Taking this together with the absent artemisinin plasma concentrations, we conclude that any putative doses of artemether-lumefantrine were likely taken several weeks before CHMI. Although we solicited for history of antimalarial use during the screening visit and excluded volunteers who reported recent treatment, volunteers may have previously taken medication with little or no explanation from medical staff. We therefore believe our findings are due to inadvertent or forgotten previous use of antimalarial drugs rather than recent surreptitious antimalarial use to influence outcomes of CHMI.

In some volunteers Kilifi qPCR results were negative throughout. The Kilifi assay has an analytical sensitivity of 20 estimated parasites per mL. Using a 0.5 or 1 mL sample, the UW qRT-PCR and the MU qPCR assays are both more sensitive than the Kilifi qPCR to parasite densities below 20 parasites per mL (15, 22). Using these more sensitive assays, 6 of 8 volunteers who were considered qPCR negative in Kilifi were found to have low-level parasitemia by UW or MU assays. If we had assessed all 33 Kilifi qPCR volunteers with UW and MU assays, we might expect the negative qPCR rate to be reduced by a similar proportion from 23% to 6%. Repeated assays of increased blood sample volumes would likely further increase the sensitivity for detecting low density parasitemia (15, 22). However, as there were 2 of 8 volunteers for whom all the samples analyzed were negative with all 3 qPCR assays, we cannot exclude the possibility of sterile pre-erythrocytic immunity in some volunteers. Furthermore, that parasites were undetectable in some volunteers immediately after emergence from the liver (i.e., 8.5–10.5 days after CHMI) and then subsequently detectable suggests a low liver-to-blood inoculum (31), which would indicate a partial role for pre-erythrocytic–stage immune responses, albeit not necessarily sterile protection. Nevertheless, the suppression of parasite growth by host immunity is profound. Further studies are warranted and will include an analysis of parameters describing the PCR data (e.g., growth rates, time to reaching treatment thresholds, mean densities) to identify those parameters most closely reflecting host immunity and an analysis of potential biomarkers of host immunity.

In conclusion, we have shown CHMI studies to be safe in a malaria-endemic location. Past exposure to malaria in an adult population leads to a range of outcomes in CHMI studies in malaria-endemic regions. *P*. *falciparum* shows marked antigenic diversity, which results in parasites evading host immunity (32). Our data suggest that sufficiently cross-reactive blood-stage immunity can be acquired on exposure to East African parasites to clear qPCR-detectable parasitemia from a phylogenetically distant West African parasite (i.e., NF54).

## Methods

### Study design and volunteer population.

The study was open, unblinded, and nonrandomized. The protocol has been published previously (13). All volunteers received an intravenous injection (DVI) of 3.2 × 10^3^ PfSPZ of the West African NF54 strain (Sanaria PfSPZ Challenge, i.e., aseptic, purified, cryopreserved PfSPZ). Volunteers were recruited from differing malaria-endemic regions in Kenya: Ahero in Western Kenya (moderate-to-high transmission at community age–adjusted parasite rates [P*f*PR] of 40%); Kilifi South (moderate transmission, currently at a P*f*PR of 20% but historically at a P*f*PR of 40%); and Kilifi North on the Kenyan Coast (low-to-no malaria transmission at present but historically at a P*f*PR of 25%; ref. 33).

We included data from a previous study based in Nairobi where there is no malaria transmission (7), although volunteers may have been exposed during previous residence or childhood elsewhere in Kenya. The Nairobi CHMI study was conducted in a total of 28 volunteers with PfSPZ administered via the intramuscular route with doses of 125,000 (*n =* 20), 75,000 (*n =* 4), or 25,000 (*n =* 4) PfSPZ. In the Kilifi study, real-time qPCR results were used for malaria diagnosis, whereas criteria for malaria diagnosis in Nairobi were based on thick-blood film microscopy with retrospective analysis of qPCR results. A positive thick film corresponds to a qPCR quantification of approximately 50 parasites per μL (14).

### Study enrollment and administration of Sanaria PfSPZ Challenge.

For the current study in Kilifi, following recruitment and informed consent procedures, volunteers went through a screening process to determine their health status and past exposure to malaria. Furthermore, anti-schizont antibodies, as previously described (7), were measured at screening to ensure that the enrolled volunteers would include a range of anti-schizont antibody titers. This was done to ensure equal enrollment from each of the 3 tertiles of anti-schizont antibody titers identified at screening. A dose of 3.2 × 10^3^ PfSPZ was administered by DVI in 0.5 mL through a 25-gauge needle over several seconds, after which volunteers were monitored for blood parasitemia by qPCR to determine parasite growth. Volunteers were enrolled in 3 successive cohorts in 2016, 2017, and 2018.

### Safety monitoring.

All volunteers were monitored for any adverse events, solicited and unsolicited, for the duration of CHMI. Signs and/or symptoms of malaria were assessed and recorded. Abnormal laboratory findings were graded for severity based on population sex-specific adapted reference ranges (Supplemental Table 1). These were categorized either as “low” or “high,” where “low” referred to values below to the lower normal limit range and “high” to values above the upper normal limit range. In keeping with the study data tabulation model implementation guide for human clinical trials, the day of injection/inoculation was defined as day 1 (C1) and not day 0 (C0).

### qPCR for parasite detection.

In the Kilifi study, real-time qPCR results were used for endpoint criteria. Endpoint criteria were considered met and antimalarial treatment was given when (a) parasitemia reached 500 parasites per μL; (b) clinically important signs and/or symptoms were observed, with any evidence of malaria parasites by blood film positivity; or (c) at day 22 (21 days after infection). For criteria b, thick- and thin-blood films were prepared when requested by the assessing clinicians and made from whole blood by experienced microscopists who examined 100 high-power fields. In Nairobi, endpoint criteria were met when thick-blood film microscopy was positive with retrospective analysis of qPCR results. A positive thick film corresponds to a qPCR quantification of approximately 50 parasites per μL (14).

For parasite detection, a sensitive high-volume qPCR assay, detecting the *18S* ribosomal RNA *P*. *falciparum* gene, was used in real time where 500 μL whole venous blood was collected twice every day from day 8 to day 15 after infection and then once every day from day 16 to day 22 after infection (13). 500 μL venous blood samples were used to extract DNA using an automated DNA extraction and purification method (QIAsymphony platform, Qiagen) according to the manufacturer’s instructions. DNA was eluted in 100 μL DNAse free water/elution buffer, from which 13.5 μL was used to amplify the 18S ribosomal RNA gene by qPCR in triplicates in a hydrolysis probe assay using primers and probes previously described (34). The PCR cycling conditions were carried as previously described (34) using the Applied Biosystems 7500 real-time PCR system. Nontemplate control was used as a negative control (in triplicate wells) with parasite quantification against known cultured parasite standards comprising 6 serial dilutions of extracted DNA also run in triplicate. The theoretical sensitivity of this assay is 5 parasites/mL (i.e., [1000 μL/(13.5 μL × 3/100 μL)] × 500 μL), but based on serial dilutions and comparison with negative controls, we consider the actual sensitivity to be 20 parasites/mL.

The final result was the geometric mean of the 3 replicates (34). Cultured parasite standards were produced in 3 different batches across the 3 successive enrolled cohorts. We therefore retested selected samples from each CHMI cohort (2016, 2017, and 2018 – representative of samples with no parasites and low, and high parasitemia) against a final set of standards and normalized quantities against a WHO standard (35). After completion of the study, a subset of 1 mL whole blood samples was also shared with external laboratories at MU and UW for their analytically sensitive qPCR and RT-PCR assays, respectively (16, 22). The limit of detection of these assays was 10 parasites per mL for the MU qPCR assay (22) and 1 parasite per mL for the UW qRT-PCR assay (15).

### Antimalarial drug detection.

Antimalarial drug concentrations were measured in all enrolled volunteers retrospectively at 2 time points, the day before challenge (C-1) and 8 days after challenge (C+8). Plasma samples were sent to 2 independent laboratories to determine plasma concentrations, Strathmore University, Nairobi, Kenya, and Mahidol Oxford Tropical Medicine Research Unit (MORU), Bangkok, Thailand. Artemether and dihydroartemisinin concentrations were measured at MORU; sulfadoxine, pyrimethamine, and chloroquine at Strathmore University; and lumefantrine and desbutyl-lumefantrine at both laboratories. Antimalarial drug concentrations were measured by liquid chromatography–tandem mass spectrometry using previously published procedures (36) and adapted from the previously reported high-performance liquid chromatography method (37).

### MSP2 genotyping.

MSP2 genotyping and antimalarial drug detection was carried out. MSP2 genotyping was performed on blood samples collected on the day of treatment initiation for all volunteers regardless of parasite outcome during CHMI. This was done by a nested PCR, with amplification specific for FC27 and IC/3D7 allelic types, with fragment size analysis by capillary electrophoresis as previously described (38).

### Statistics.

The sample size for the study was based on considering analysis of outcome of CHMI in a multivariable model considering parameters other than PCR outcome, assuming an *r*^2^ value of 0.3, with a power of 80% to detect a single variable to account for 0.15 of the variability in parasite growth. Nonparametric correlation test and Spearman’s rank correlation were used for comparisons. All analyses were performed using STATA (version 15.1, release 15; StataCorp). All tests were performed at 5% significance level, where *P <* 0.05 was considered significant.

### Study approval.

The study was conducted at the Kenya Medical Research Institute-Wellcome Trust Research Programme in Kilifi, Kenya, and received ethical approval from the Kenya Medical Research Institute Scientific and Ethics Review Unit (KEMRI/SERU/CGMR-C/029/3190) and the University of Oxford Tropical Research Ethics Committee (OxTREC 2-16). The study is registered on ClinicalTrials.gov (NCT02739763) and was conducted based on good clinical practice and under the principles of the Declaration of Helsinki.

## Author contributions

MCK, PFB, and CHMI-SIKA Study Team members contributed to the study design. Protocol development was performed by MCK, PN, and PFB. Conduct of the clinical trial was performed by PN, MH, JM, ON, and CHMI-SIKA Study Team members. Experiments and data acquisition were conducted by MCK, DK, JMN, and CHMI-SIKA Study Team members. EO and CHMI-SIKA Study Team members acquired and curated data. CHMI-SIKA Study Team members were involved in manufacture of Sanaria PfSPZ Challenge. MCK, EO, and PFB analyzed the data. MCK and PFB wrote the manuscript. All the members of the CHMI-SIKA study team read and reviewed the manuscript.

## Supplementary Material

Supplemental data

Supplemental data 1

Supplemental data 2

Trial reporting checklists

ICMJE disclosure forms

## Figures and Tables

**Table 1:**
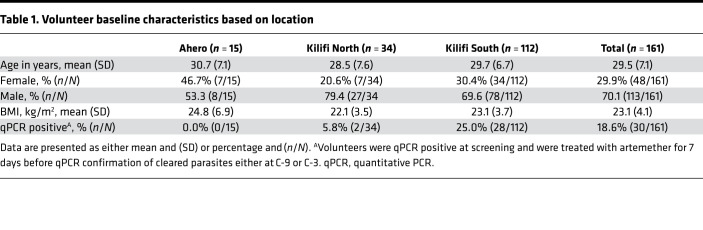
Volunteer baseline characteristics based on location

**Table 2:**
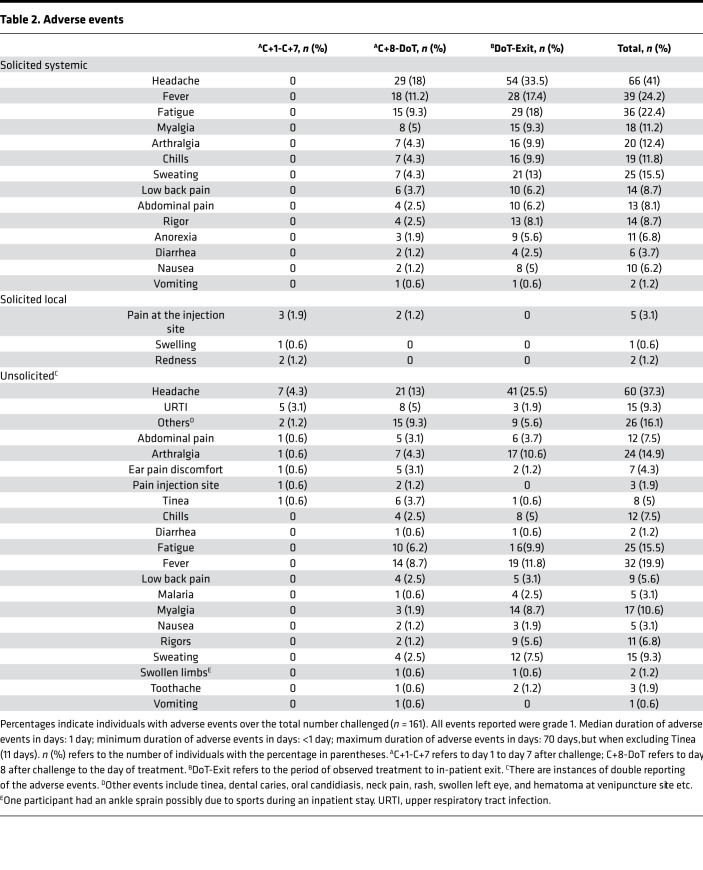
Adverse events

**Table 3:**
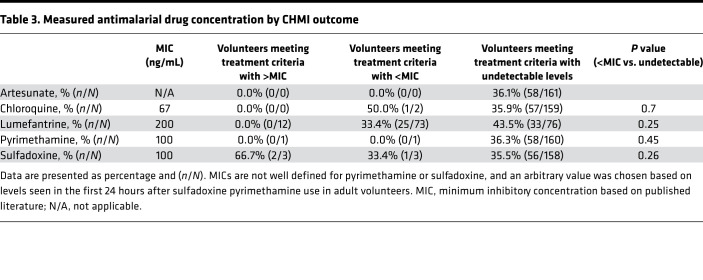
Measured antimalarial drug concentration by CHMI outcome

**Table 4:**
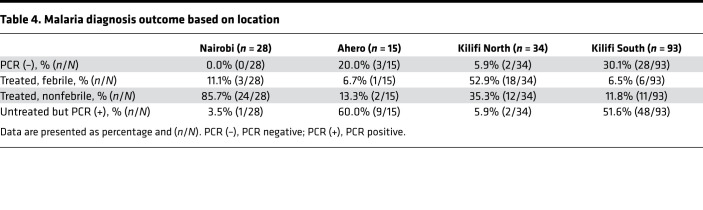
Malaria diagnosis outcome based on location

## Version 1 07/15/2021

In-Press Preview

## Version 2 09/08/2021

Electronic publication
