# Supplementary material for: Safety and PCR monitoring in 161 semi-immune Kenyan adults following controlled human malaria infection
Source: JCI Insight. 2021 Sep 8;6(17):e146443. doi: 10.1172/jci.insight.146443 (PMC8492329; doi:10.1172/jci.insight.146443)
Supplement: Supplemental data [file jciinsight-6-146443-s133.pdf]

## Supplementary Data

## Supplementary Table 1. Laboratory reference ranges with grading criteria

| Parameter | Reference ranges | Grade 1 (Mild) | Grade 2 (Moderate) | Grade 3 (Severe) | Grade 4 (Potentially life threatening) |
|---|---|---|---|---|---|
| HAEMATOLOGY – Adult Male | | | | | |
| Hemoglobin-Hb (Low) g/dL | 10.6-17.0 | 10.0-<10.6 | 9-<10.0 | 7.0-< 9.0 | <7.0 with clinical signs |
| Lymphocytes (Low) (×10^3 cells/µl) | 1.2-3.5 | 0.6 - < 1.2 | 0.5-<0.6 | 0.35-<0.5 | < 0.35 |
| Neutrophils (× 10^3 cells/µl) | 0.9-7.7 | 0.8-<0.9 | 0.6-0.799 | 0.4-0.599 | <0.4 |
| WBC (low) (× 10^3cells/µl) | 3.0-10.1 | 2.0-<3.0 | 1.5-1.9 | 1.0-1.4 | <1 |
| PLT (× 10^3 cells/µl) | 139-398 | 100-<139 | 50-<100 | 25-<50 | <25 |
| HAEMATOLOGY – Adult Female | | | | | |
| Hb (Low) g/dL | 8.0-14.9 | 7-<8 | 6-<7 | 5- 6 | <5 with clinical signs |
| Lymphocytes (× 10^3cells/µl) | 1.3 – 3.5 | 0.6-<1.3 | 0.5-<0.6 | 0.35-<0.5 | < 0.35 |
| Neutrophils (× 10^3 cells/µl) | 1.1-5.3 | 0.8-1.1 | 0.6-0.799 | 0.4-0.599 | <0.4 |
| WBC (× 10^3 cells/µl) | 3.3-8.6 | 2.0-<3.3 | 1.5-1.9 | 1.0-1.4 | < 1 |
| PLT (× 10^3 cells/µl) | 166-456 | 100-< 166 | 50-<100 | 25-<50 | <25 |
| BIOCHEMISTRY – Adult Male | | | | | |
| Sodium (mmol/L) Low | 135-145 | 130 to < 135 | 125-<130 | 121-<125 | ≤ 120 |
| Sodium (mmol/L) High | 135-145 | 146-150 | 150-154 | 154-160 | ≥160 |
| Potassium (mmol/L) Low | 3.5-4.5 | 3.0-<3.4 | 2.5-<3.0 | 2.0-<2.5 | <2.0 |
| Potassium (mmol/L) High | 3.5-4.5 | 4.5-<6.0 | 6.0-<6.5 | 6.5-<7.0 | ≥7.0 |
| Urea (mmol/L) – Do not grade | 2.1-7.1 | | | | |
| Creatinine (µmol/L) High | 44-111 | >111-144 1.1-1.3 X ULN | >144-199 >1.3-1.8 X ULN | 199-<389 >1.8-<3.5 X ULN | ≥ 388 ≥3.5 X ULN |
| ALT (IU/L) | 11-80 | >80-<200 1.25-<2.5 X ULN | 200-<400 2.5-<5.0 ULN | 400-<800 5.0-<10.0 ULN | ≥ 800 ≥10.0 X ULN |
| BIOCHEMISTRY – Adult Female | | | | | |
| Sodium mmol/ (Low) | 135-145 | 130-<135 | 125-<130 | 121-<125 | ≤ 120 |
| Sodium mmol/L (High) | 135 145 | >145-<150 | 150-<154 | 154-<160 | ≥ 160 |
| Potassium mmol/L | 3.4-4.4 | 3.0-<3.4 | 2.5-<3.0 | 2.0-< 2.5 | < 2.0 |
| Urea (mmol/L) – Do not grade | 2.1-7.1 | | | | |
| Creatinine (µmol/L) (High) | 37-92 | >92-<119 1-1.3 X ULN | >119-<166 >1.3-1.8 X ULN | >166-322 >1.8-<3.5ULN | ≥ 322 ≥ 3.5 X ULN |
| ALT (IU/L) | 8-55 | >55-<138 1< 2.5 ULN | 138-<275 2.5-<5.0 x ULN | 275-<550 5.0-10 X ULN | ≥ 550 ≥10.0 X ULN |

‘Low’ refers to values below to the lower normal limit range; ‘High’ refers to values above the upper normal limit range

**Supplementary Table 2. Overall Concomitant Medication Use based on location**

| | Kilifi North (N=34) | Kilifi South (N=112) | Ahero (N=15) | Total (N=161) |
|---|---|---|---|---|
| Amoxycillin | 3 (8.8) | 11 (9.8) | 2 (13.3) | 16 (9.9) |
| Amoxicillin/Clavulanate | 0 | 1 (0.9) | 0 | 1 (0.6) |
| Antacid | 0 | 4 (3.6) | 0 | 4 (2.5) |
| Antihistamine | 2 (5.9) | 4 (3.6) | 2 (13.3) | 8 (5) |
| Ceftriaxone | 0 | 1 (0.9) | 0 | 1 (0.6) |
| Clotrimazole | 4 (11.8) | 9 (8) | 0 | 13 (8.1) |
| Diclofenac | 0 | 1 (0.9) | 0 | 1 (0.6) |
| Diclofenac Gel | 0 | 7 (6.3) | 0 | 7 (4.3) |
| Ibuprofen | 10 (29.4) | 13 (11.6) | 1 (6.7) | 24 (14.9) |
| Nystatin Oral Drops | 1 (2.9) | 1 (0.9) | 1 (6.7) | 3 (1.9) |
| ORS | 0 | 3 (2.7) | 0 | 3 (1.9) |
| Paracetamol | 21 (61.8) | 39 (34.8) | 11 (73.3) | 71 (44.1) |

Data are presented as n (%) or otherwise specified. ORS, oral rehydration salts.

**Supplementary Table 3 Abnormal laboratory result summaries and severity grades**

| | Grade | C-1* | 8 to DoD* | DoD to Exit* | LTFU** |
|---|---|---|---|---|---|
| ALT (Low) | 1 | 0 | 1 (0.6) | 2 (1.2) | 0 |
| ALT (High) | 1 | 2 (1.2) | 45 (28) | 34 (21.1) | 0 |
| | 2 | 0 | 7 (4.3) | 5 (3.1) | 0 |
| Creatinine (Low) | 1 | 0 | 1 (0.6) | 1 (0.6) | 0 |
| Creatinine (High) | 1 | 9 (5.6) | 33 (20.5) | 23 (14.3) | 3 (8.6) |
| Bilirubin (Low) | | 5(3.1) | 18(11.2) | 10(6.2) | 2(5.7) |
| Bilirubin (High) | | 3(1.9) | 2(1.2) | 1(0.6) | 1(2.9) |
| Albumin (Low) | | 3(1.9) | 10(6.2) | 9(5.6) | 0 |
| Albumin (High) | | 21(13) | 16(9.9) | 25(15.5) | 0 |
| Potassium (Low) | 1 | 22 (13.7) | 21 (13.0) | 16 (9.9) | 8 (5.0) |
| | 2 | 2 (1.2) | 0 | 0 | 0 |
| Potassium (High) | 1 | 4 (2.5) | 16 (9.9) | 9 (5.6) | 0 |
| Sodium (Low) | 1 | 0 | 25 (15.5) | 33 (20.5) | 11 (31.4) |
| Urea (Low) | | 6 (3.7) | 2 (1.2) | 3 (1.9) | 1 (2.9) |
| Hb (Low) | 1 | 1 (0.6) | 1 (0.6) | 0 | 0 |
| | 3 | 1 (0.6) | 0 | 0 | 0 |
| Hb (High) | | 4 (2.5) | 2 (1.2) | 4 (2.5) | 0 |
| Platelets (Low) | 1 | 7 (4.3) | 11 (6.8) | 20 (12.4) | 8 (24.2) |
| | 2 | 0 | 1 (0.6) | 2 (1.2) | 1 (3) |
| | 3 | 0 | 1 (0.6) | 1 (0.6) | 0 |
| | 4† | 0 | 1 (0.6) | 1 (0.6) | 0 |
| Platelets (High) | | 6 (3.7) | 5 (3.1) | 6 (3.7) | 0 |
| WBC (Low) | 1 | 1 (0.6) | 4 (2.5) | 6 (3.7) | 1 (3) |
| WBC (High) | 0 | 4 (2.5) | 18 (11.2) | 19 (11.8) | 0 |

C-1 refers to a day before challenge; 8-DoT refer to days after challenge; DoT-Exit refers to period of observed treatment to in-patient exit; LTFU refers to long term follow up; *Percentages expressed over total subjects challenged (N=161); **Percentages expressed over number of subjects followed up for each respective parameter – chemistry (N=35) and haematology (N=33); Duration to normal ALT levels: median 14 days (min 2 days and max 67 days); †Grade 4 low platelet reading was 22x10^3 cells/µl on days 22 and 24 with duration to normal levels: median 11 days (min 2 days and max 71 days). 'Low' refers to values below to the lower normal limit range; 'High' refers to values above the upper normal limit range

**Supplementary Table 4: qPCR outcomes by location after exclusion of all volunteers with any detectable lumefantrine levels**

| | Nairobi (N=28) | Ahero (N=7) | Kilifi North (N=27) | Kilifi South (N=40) |
|---|---|---|---|---|
| PCR (-) | 0% (0/28) | 14.3% (1/7) | 7.4% (2/27) | 17.5% (7/40) |
| Treated, febrile | 10.7% (3/28) | 14.3% (1/7) | 51.9% (14/27) | 7.5% (3/40) |
| Treated, non-febrile | 85.7% (24/28) | 14.3% (1/7) | 37.0% (10/27) | 5.0% (2/40) |
| Untreated but PCR (+) | 3.6% (1/28) | 57.1% (4/7) | 3.7 (1/27) | 70.5% (28/40) |

Data are presented as % and (n/N) or otherwise specified. PCR (-) refers to PCR negative; PCR (+) refers to PCR positive.

**Supplementary figure legends**

**Supplementary Figure 1 Plasma ALT concentration over time based on gender.**

Plasma samples one day before challenge (C-1), days 10 or 22 or day of treatment (DoT), and day 36 after challenge including a sample taken after late follow up were analysed for ALT concentrations (IU/L) based on gender. Volunteers (N=142) were inoculated on C1 and followed over time. LFTU refers to long term follow up.

**Supplementary Figure 2 Anti-malarial drug concentrations based on treatment outcome.**

Plasma samples taken from volunteers one day before challenge (C-1) and seven days after challenge (C+8) were analysed for the anti-malarials: (A) chloroquine; (B) lumefantrine; (C) sulphadoxine; and (D) pyrimethamine. The red horizontal line represents the minimum inhibitory concentration for therapeutic efficacy for each respective drug. Treatment Required refers to whether volunteers were treated before C+22 (Yes) or on C+22 (No). Artemether and dihydroartemisinin levels were all undetectable.

**Supplementary Figure 3 Individual qPCR results based on volunteer outcome and location**

Blood samples from C+8 to C+22 after CHMI were used for qPCR from: (A) Nairobi – N=28; (B) Kilifi North – N=34; (C) Kilifi South – N=93; and (D) Ahero – N=15. Parasitaemia was determined by asexual *18S* ribosomal RNA gene qPCR for each volunteer (limit of detection 20 parasites/ml). Blue lines represent individuals who required treatment and reached treatment threshold (reached DoT); green lines represent individuals who did not meet criteria for treatment threshold but were qPCR positive; orange lines represent individuals who were qPCR negative throughout monitoring; and red dot denotes individuals were febrile and met treatment

criteria. Shown are each of the 142 volunteers with their respective subject identification numbers.

**Supplementary Figure 4 Individual qPCR results based on volunteer outcome and cohort**

Blood samples from C+8 after inoculation were used to determine estimated parasite density from each volunteer enrolled into the cohorts: (A) 2016 – N=36; (B) 2017 – N=53; and (C) 2018 – N=93. Parasitaemia was determined by asexual *Plasmodium 18S* ribosomal RNA gene qPCR for each volunteer. Blue lines represent individuals who required treatment and reached treatment threshold (reached DoT); green lines represent individuals who did not meet criteria for treatment threshold but were qPCR positive; orange lines represent individuals who were qPCR negative throughout monitoring; and red dot denotes individuals were febrile and met treatment criteria. Shown are each of the 142 volunteers with their respective subject identification numbers.

**Supplementary Figure 5 Matrix correlation for qPCR and qRT-PCR assay comparisons**

Blood samples from 20 volunteers from the 2018 cohort representing six time points (C+8 to C+10.5) after inoculation were used to estimate parasitaemia using *Plasmodium 18S* rRNA gene qPCR assays from Kilifi and Mahidol University (MU) and *18S* rRNA qRT-PCR from the University of Washington (UW). Shown are Spearman correlation comparing the respective assays. Units on both axes are estimated parasites/mL. Negative results are displayed at the LOD for each assay (Kilifi 20 parasites/mL, UW 1 parasite/mL, MU 10 parasites/mL).

**Supplementary Figure 6 Individual qPCR and qRT-PCR post-challenge results**

Blood samples from 20 volunteers from the 2018 cohort representing six time points (C+8 to C+10.5) after inoculation were used to determine parasitaemia using the *Plasmodium 18S*

rRNA gene qPCR assays from Kilifi (green) and Mahidol (orange), and the *Plasmodium 18S* rRNA qRT-PCR from Washington (blue). Shown are each of the 20 volunteers with their respective subject identification numbers. Negative results are shown at 10 (Kilifi), 0.05 (MU) and 0.005 (UW) parasites/ml in order to graph separate lines.

**Supplementary Figure 7 ALT concentration over time based on qPCR status**

Plasma samples a day before challenge (C-1), Days 10 or 22 or day of treatment (DoT), and Day 36 after challenge including a sample taken after late follow-up were analysed for ALT concentrations (IU/L) based on qPCR status. Volunteers were inoculated on C1 and followed over time and exited the study at C+36.

**Data availability**

Volunteer data will be deposited into the KWTRP Harvard Dataverse repository and made available including data dictionaries after de-identification of the volunteer data that represent the results reported in this article. The data will be available to researchers who will need to submit proposals to dgc@kemri-wellcome.org to gain access to the data following a signed data access agreement. The study protocol, informed consent forms, and all other associated documents have been previously published.

**Supplemental Acknowledgements: Members of the CHMI-SIKA Study Team**

| First Initials | Surname |
|---|---|
| A. I. | Abdi[1] |
| Y. | Abebe[2] |
| A. | Audi[3] |
| P. | Bejon[1,4] |
| P. C. | Bull[5] |
| P. C. | Che[1] |
| K. P. | Cruz[6] |
| Z. | de Laurent[1] |
| S. H. | Hodgson[7] |
| S. L. | Hoffman[2] |
| E.R. | James[2] |
| I. | Jao[1] |
| D. | Kamuya[1] |
| S. | Kariuki[1] |
| N. | Kibinge[1] |
| R. | Kimathi[1] |
| S. | Kinyanjui[1,4,8] |
| C. | Kivisi[8] |
| N. | Koskei[3] |
| M. | Imwong[9] |
| J. | Makale[1] |
| K. | Marsh[1,4] |
| V. | Marsh[1,4] |
| K. S. | Mohammed[1] |
| M. | Mosobo[1] |
| S. C. | Murphy[6] |
| J. | Musyoki[1] |
| M. | Muthui[1] |
| J. | Mwacharo[1] |
| K. | Mwai[1,10] |
| D. | Mwanga[1] |
| I. | Nkumama[1,11] |
| F.M | Ndungu[1,4] |
| M. | Njue[1] |
| G. | Nyangweso[1] |
| D. | Odera[1,11] |
| B. | Ogutu[3,12] |
| F. | Olewe[3,12] |
| J. | Oloo[3] |
| D. | Omuoyo[1] |
| J. | Ongecha[3,12] |
| M. O. | Ongas[3,12] |
| M. | Ooko[1] |
| N. | Smith[6] |
| F. | Osier[1,11] |

| First Initials | Surname |
| --- | --- |
| T. L. | Richie[2] |
| A. M. | Seilie[6] |
| J. | Shangala[1] |
| B. K. L. | Sim[2] |
| B. | Sriburin[13] |
| J. | Tarning[4,13] |
| J. | Tuju[1] |
| J. | Wambua[1] |
| T. N. | Williams[1,14] |
| M. | Winterberg[4,13] |

Affiliations

[1]KEMRI-Wellcome Trust Research Programme, Kilifi, Kenya

[2]Sanaria Inc., Rockville, MD, USA

[3]Centre for Clinical Research, Kenya Medical Research Institute, Kisumu, Kenya

[4]Centre for Tropical Medicine and Global Health, Nuffield Department of Medicine, University Oxford, Oxford, UK

[5]Department of Pathology, University of Cambridge, Cambridge, UK

[6] Departments of Laboratory Medicine and Microbiology and Center for Emerging and Re-emerging Infectious Diseases, University of Washington, Seattle, Washington, USA

[7]The Jenner Institute, University of Oxford, Oxford, UK.

[8]Pwani University, P. O. Box 195-80108, Kilifi, Kenya

[9]Faculty of Tropical Medicine, Department of Molecular Tropical Medicine and Genetics, Mahidol University, Bangkok, Thailand

[10]Epidemiology and Biostatistics Division, School of Public Health, University of the Witwatersrand, Johannesburg, South Africa.

[11]Centre for Infectious Diseases, Heidelberg University Hospital, Heidelberg, Germany

[12]Center for Research in Therapeutic Sciences, Strathmore University, Nairobi, Kenya

[13]Mahidol-Oxford Tropical Medicine Research Unit, Mahidol University, Bangkok, Thailand

[14]Department of Medicine, Imperial College, London, UK
